# Unique epithelial expression of S100A calcium binding protein A7A in the endometrium at conceptus implantation in pigs

**DOI:** 10.5713/ajas.18.0920

**Published:** 2019-02-09

**Authors:** Soohyung Lee, Hwanhee Jang, Inkyu Yoo, Jisoo Han, Wonchul Jung, Hakhyun Ka

**Affiliations:** 1 Department of Biological Science and Technology, Yonsei University, Wonju 26493, Korea

**Keywords:** Pig, Uterus, Endometrium, S100A7A

## Abstract

**Objective:**

S100A7A, a member of the S100 protein family, is involved in various biological processes, including innate immunity, antimicrobial function, and epithelial tumorigenesis. However, the expression and function of S100A7A in the endometrium during the estrous cycle and pregnancy are not well understood in pigs. Therefore, this study determined the expression and regulation of S100A7A at the maternal-conceptus interface in pigs.

**Methods:**

We obtained endometrial tissues from pigs throughout the estrous cycle and pregnancy, conceptus tissues during early pregnancy, and chorioallantoic tissues during mid- to late pregnancy and analyzed the expression of *S100A7A* in these tissues. We also determined the effects of steroid hormones, estradiol-17β (E_2_) and progesterone, and interleukin-1β (IL1B) on *S100A7A* expression in endometrial tissues.

**Results:**

We found that *S100A7A* was expressed in the endometrium during the estrous cycle and pregnancy in a pregnancy status- and stage-dependent manner and was localized to endometrial luminal epithelial (LE) and superficial glandular epithelial cells with strong intensity in LE cells on day 12 of pregnancy. Early stage conceptuses and chorioallantoic tissues from day 30 to term pregnancy also expressed *S100A7A*. The expression of *S100A7A* was increased by E_2_ and IL1B in endometrial tissues.

**Conclusion:**

*S100A7A* was expressed at the maternal-conceptus interface at the initiation of implantation in response to conceptus-derived estrogen and IL1B and could be a unique endometrial epithelial marker for conceptus implantation in pigs. These findings provide an important insight into the understanding of conceptus-endometrial interactions for the successful establishment of pregnancy in pigs.

## INTRODUCTION

The implantation process is a series of complex interactions between the endometrium and the conceptus (embryo/fetus and associated extra-embryonic membranes) during early pregnancy. In pigs, the conceptus undergoes rapid elongation, from spherical to filamentous forms, between days 10 and 12 of pregnancy, which corresponds to the time when the conceptus initiates implantation to the endometrium [1]. At this time, the conceptus secretes estrogen and interleukin-1β2 (IL1B2) [2–4]. In pigs, estrogen secreted by the conceptus acts as a pregnancy recognition signal by redirecting the endometrial secretory pattern of prostaglandin F_2α_ (PGF_2α_) from vasculature to uterine lumen to prevent the corpus luteum (CL) from undergoing luteolysis [1]. Estrogen also induces the expression of many endometrial genes, including aldo-keto reductase 1B1 (*AKR1B1*), fibroblast growth factor 7 (*FGF7*), interferon alpha and beta receptor subunit 2 (*IFNAR2*), lysophosphatidic acid receptor 3 (*LPAR3*), S100 calcium binding protein G (*S100G*), secreted phosphoprotein 1 (*SPP1*), signal transducer and activator of transcription 1 (*STAT1*), and transient receptor potential cation channel subfamily V member 6 (*TRPV6*) [2–4]. IL1B regulates the expression of endometrial genes for prostaglandin synthesis and transport by regulating the endometrial expression of PG-endoperoxide synthase 1 (*PTGS1*), *PTGS2*, *AKR1B1*, ATP-binding cassette sub-family C member 4 (ABCC4), ABCC9, solute carrier organic anion transporter family member 2A1 (*SLCO2A1*), *SLCO4C1*, and *SLCO5A1* [5,6]. These molecules induced by estrogen and IL1B in the endometrium play critical roles in the establishment and maintenance of pregnancy in pigs [4].

Among many genes expressed in the endometrium at the time of implantation, we have shown that the expression of S100A calcium binding protein A7A (*S100A7A*) was differentially expressed in the endometrium on day 12 of pregnancy compared to that on day 12 of the estrous cycle in pigs [7]. S100A7A, also known as S100A15, is a member of the S100 protein family and is characterized by two calcium binding sites with a helix-loop-helix (EF-hand) motif [8]. S100A7A is also called koebnerisin because it was first identified in koebnerized psoriatic skin from psoriatic patients [9]. In humans, *S100A7A* is expressed by various types of skin cells, including keratinocytes and dendritic cells [10]. The expression of *S100A7A* is restricted to the proliferative basal cell layer in normal skin but to the basal and granular layers in non-lesional psoriatic skin [9,11]. It has been shown that S100A7A acts as an antibacterial protein by reducing survival of *Escherichia coli* in skin [12] and shows chemotactic activity for granulocytes and monocytes but not for lymphocytes in human skin [10]. The expression of *S100A7A* in human skin is up-regulated by calcium, IL1B, and Th1 cytokines, tumor necrosis factor-α, and interferon-γ (IFN-γ), suggesting that S100A7A is involved in the inflammatory response [11]. Although the expression and function of S100A7A are well studied in humans, the expression, regulation, and function of S100A7A in the endometrium during the estrous cycle and pregnancy are not well understood in pigs.

Therefore, to better understand the role of S100A7A at the maternal-conceptus interface in pigs, we determined: i) the expression of *S100A7A* in the endometrium during the estrous cycle and pregnancy, in the conceptus of early-stage pregnancy, and in chorioallantoic tissues during mid- to term pregnancy, ii) localization of *S100A7A* in the endometrium, and iii) effects of steroid hormones and IL1B on *S100A7A* expression in endometrial tissues.

## MATERIALS AND METHODS

### Animals and tissue preparation

All experimental procedures involving animals were conducted in accordance with the Guide for Care and Use of Research Animals in Teaching and Research and approved by the Institutional Animal Care and Use Committee of Yonsei University and the National Institute of Animal Science. Sexually mature crossbred female gilts were assigned randomly to either cyclic or pregnant status. The reproductive tracts of the gilts were obtained immediately after slaughter on day 0 (the onset of estrous), 3, 6, 9, 12, 15, or 18 of the estrous cycle and day 12, 15, 30, 60, 90, or 114 of pregnancy (n = 3 – 6 gilts/d/status). Pregnancy was confirmed by the presence of apparently normal spherical to filamentous conceptuses in uterine flushings on days 12 and 15 and the presence of embryos and placenta on the later days of pregnancy. Chorioallantoic tissues were obtained from days 30, 60, 90, and 114 of pregnancy (n = 3 – 4 gilts/d). Endometrium, dissected free of myometrium, was collected from the middle portion of each uterine horn, snap-frozen in liquid nitrogen, and stored at −80°C prior to RNA extraction. For *in situ* hybridization, cross-sections of endometrium were fixed in 4% paraformaldehyde in phosphate-buffered saline (PBS) (pH 7.4) for 24 h and then embedded in paraffin as previously described [13].

### Total RNA extraction and porcine *S100A7A* cDNA cloning

Total RNA was extracted from endometrial and conceptus tissues using TRIzol reagent (Invitrogen Life Technology, Carlsbad, CA, USA), according to the manufacturer’s recommendations. The quantity of RNA was assessed spectrophotometrically, and the RNA integrity was validated following electrophoresis in 1% agarose gel.

Four micrograms of total RNA were treated with DNase I (Promega, Madison, WI, USA) and reverse transcribed using SuperScript II Reverse Transcriptase (Invitrogen, USA) to obtain cDNAs. The cDNA templates were then diluted at a 1:4 ratio with sterile water and amplified by polymerase chain reaction (PCR) using Taq polymerase (Takara Bio, Shiga, Japan). The PCR conditions and sequences of primer pairs are listed in Table 1. The PCR products were separated on 2% agarose gel and visualized by ethidium bromide staining. The identity of each amplified PCR product was verified by sequence analysis after cloning into the pCRII vector (Invitrogen, USA).

### Quantitative real-time reverse transcription polymerase chain reaction

The level of *S100A7A* expression in endometrial and chorioallantoic tissues was analyzed by real-time reverse transcription polymerase chain reaction (RT-PCR) using the Applied Biosystems StepOnePlus System (Applied Biosystems, Foster City, CA, USA) with the SYBR Green method. The Power SYBR Green PCR Master Mix (Applied Biosystems, USA) was used for PCR reactions. The final reaction volume of 20 μL included 2 μL of cDNA, 10 μL of 2X Master mix, 2 μL of each primer, and 4 μL of ddH_2_O. The PCR conditions and sequences of primer pairs are listed in Table 1. The results are reported as expression relative to that detected on day 12 of the estrous cycle or that detected in control explant tissues after normalization of the transcript amount to the endogenous porcine ribosomal protein L7, ubiquitin B, and TATA binding protein controls by the 2^−ΔΔCT^ method, as previously described [14].

### Nonradioactive *in situ* hybridization

Nonradioactive *in situ* hybridization was performed to determine the localization of *S100A7A* expression in the uterine endometrium, as previously described, with some modifications [6,15]. Sections (5 μm thick) were rehydrated through successive baths of xylene, 100% ethanol, 95% ethanol, diethylpyrocarbonate (DEPC)-treated water, and DEPC-treated PBS. Tissue sections were boiled in citrate buffer (pH 6.0) for 10 min. After washing in DEPC-treated PBS, they were digested using 5 μg/mL proteinase K (Sigma, St. Louis, MO USA) in TE buffer (100 mM Tris-HCl, 50 mM ethylenediaminetetraacetic acid, pH 7.5) at 37°C. After postfixation in 4% paraformaldehyde, tissue sections were incubated twice for 15 min each in PBS containing 0.1% active DEPC and were equilibrated for 15 min in 5× saline sodium citrate (SSC). The sections were prehybridized for 2 h at 68°C in a hybridization mix (50% formamide, 5× SSC, 500 μg/mL herring sperm DNA, 250 μg/mL yeast tRNA). Sense and antisense riboprobes for each gene were generated using partial cDNAs cloned into pCRII vectors by linearizing with appropriate restriction enzymes and labeling with digoxigenin (DIG)-UTP using a DIG RNA Labeling kit (Roche, Indianapolis, IN, USA). The probes were denatured for 5 min at 80°C and added to the hybridization mix. The hybridization reaction was carried out overnight at 68°C. Prehybridization and hybridization reactions were performed in a box saturated with a 5× SSC 50% formamide solution to avoid evaporation, and no coverslips were used. After hybridization, sections were washed for 30 min in 2× SSC at room temperature, 1 h in 2× SSC at 65°C, and 1 h in 0.1× SSC at 65°C. Probes bound to the section were detected immunologically using sheep anti-DIG Fab fragments covalently coupled to alkaline phosphatase and nitro blue tetrazolium chloride/5-bromo-4-chloro-3-indolyl phosphate (toluidine salt) as a chromogenic substrate, according to the manufacturer’s protocol (Roche, USA).

### Explant cultures

To determine the effects of steroid hormones, E_2_ and progesterone (P_4_), and IL1B on *S100A7A* expression in the endometrium, endometrial explant tissues obtained from gilts on Day 12 of the estrous cycle were cultured as previously described [13,16]. The endometrium was dissected from the myometrium and placed into warm phenol red-free Dulbecco’s modified Eagle’s medium/F-12 culture medium (DMEM/F-12; Sigma, USA) containing penicillin G (100 IU/mL) and streptomycin (0.1 mg/mL). The endometrium was minced into small pieces (2 to 3 mm^3^) with scalpel blades, and aliquots of 500 mg were placed into T25 flasks with serum-free modified DMEM/F-12 containing 10 μg/mL insulin (Sigma, USA), 10 ng/mL transferrin (Sigma, USA), and 10 ng/mL hydrocortisone (Sigma, USA). Endometrial explants were cultured immediately after mincing in the presence of ethanol (control), E_2_ (10 ng/mL; Sigma, USA), P_4_ (30 ng/mL; Sigma, USA), P_4_+E_2_, P_4_+E_2_+ICI182,780 (ICI; an estrogen receptor antagonist; 200 ng/mL; Tocris Bioscience, Ellisville, MO, USA), or P_4_+E_2_+RU486 (RU; a progesterone receptor [PGR] antagonist; 30 ng/mL; Sigma, USA) for 24 h with rocking in an atmosphere of 5% CO_2_ in air at 37°C. To determine the effect of IL1B on endometrial *S100A7A* expression, explant tissues were treated with 0, 1, 10, or 100 ng/mL IL1B (Sigma, USA) in the presence of both E_2_ (10 ng/mL) and P_4_ (30 ng/mL) at 37°C for 24 h. Explant tissues were then harvested, and total RNA was extracted for real-time RT-PCR to determine *S100A7A* mRNA level. These experiments were conducted using endometria from three gilts on day 12 of the estrous cycle, and treatments were performed in triplicate using tissues obtained from each of the three gilts.

### Statistical analysis

Data from real-time RT-PCR for *S100A7A* expression were subjected to analysis of variance using the General Linear Models procedures of SAS (Cary, NC, USA). As sources of variation, the model included day, pregnancy status (cyclic or pregnant, days 12 and 15 post-estrus), and their interactions to evaluate steady-state levels of *S100A7A* mRNA. Data from real-time RT-PCR performed to assess the effects of day of the estrous cycle (day 0, 3, 6, 9, 12, 15, and 18) and pregnancy (day 12, 15, 30, 60, 90, and 114) in the endometrium, the effects of day of pregnancy in chorioallantoic tissue (day 30, 60, 90, and 114), and the effect of IL1B dose in explant culture were analyzed by least squares regression analysis. Data from real-time RT-PCR to assess the effects of steroid hormone in explant culture were analyzed by preplanned orthogonal contrasts (control vs E_2_; control vs P_4_; P_4_ vs P_4_+E_2_; P_4_+E_2_ vs P_4_+E_2_+ICI; and P_4_+E_2_ vs P_4_+E_2_+RU). Data are presented as mean with standard error of the mean. A p-value <0.05 was considered significant.

## RESULTS

### *S100A7A* expression in the endometrium during the estrous cycle and pregnancy

**Figure 1:**
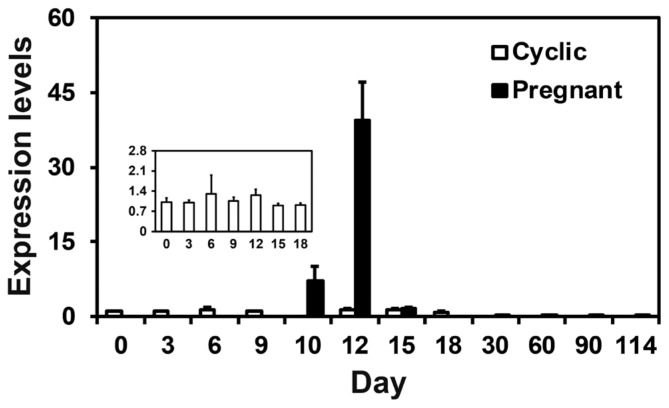
S100A calcium binding protein A7A (*S100A7A*) expression in the endometrium during the estrous cycle and pregnancy. Endometrial tissue samples from cyclic and pregnant gilts were analyzed by real-time reverse transcription polymerase chain reaction, and data are reported as expression relative to that detected on day 0 of the estrous cycle after normalization of the transcript amount to the endogenous porcine ribosomal protein L7, ubiquitin B, and TATA binding protein controls. Data are presented as mean with standard error.

We examined the relative abundance of *S100A7A* mRNA in the endometrium using real-time RT-PCR to determine whether the expression of *S100A7A* changed in the endometrium during the estrous cycle and pregnancy in pigs. As shown in Figure 1, the expression of *S100A7A* in the endometrium did not change during the estrous cycle, while it changed during pregnancy, with the greatest abundance on day 12 of pregnancy (linear effect of day, p<0.05). On days 12 and 15 post-estrus, *S100A7A* expression was affected by day (p<0.01), pregnancy status (p<0.01), and day×status (p< 0.01), and the expression of *S100A7A* was greater on day 12 of pregnancy than day 12 of the estrous cycle (p<0.05).

### *S100A7A* mRNA localization in the endometrium during the estrous cycle and pregnancy

**Figure 2:**
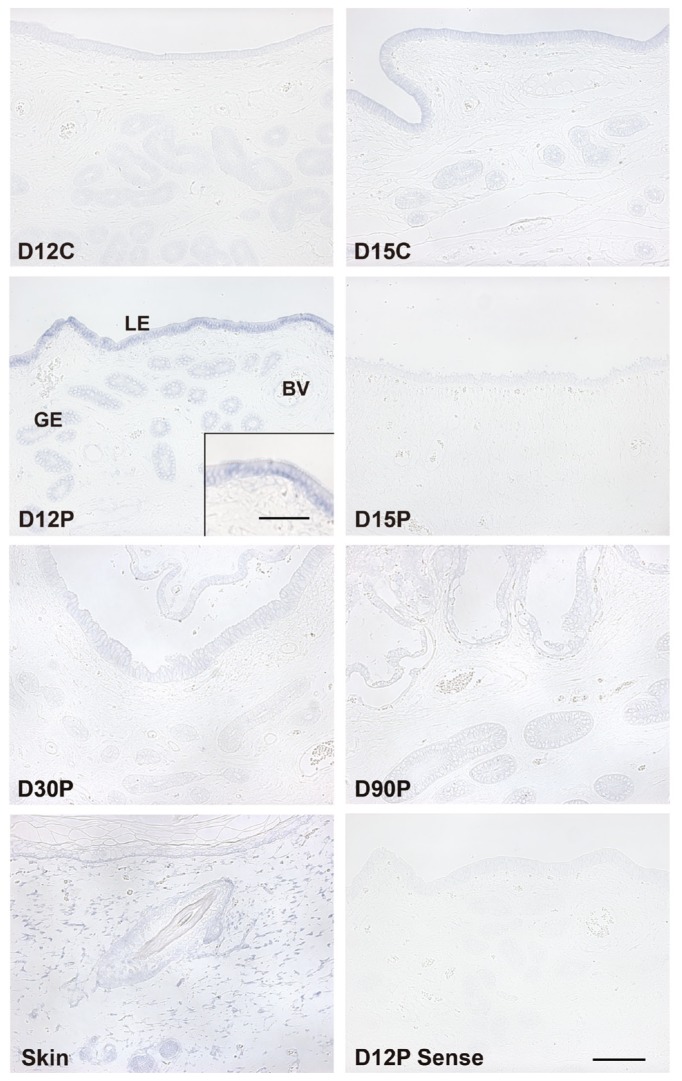
*In situ* hybridization analysis of S100A calcium binding protein A7A (*S100A7A*) expression in the endometrium during the estrous cycle and pregnancy. The expression of *S100A7A* mRNA was detected primarily in LE cells on day 12 of pregnancy. A uterine section from adult skin hybridized with a digoxigenin (DIG)-labeled antisense *S100A7A* cRNA probe is shown as a positive control, and a uterine section from day 12 of pregnancy hybridized with a DIG-labeled sense *S100A7A* cRNA probe (Sense) served as a negative control. D, day; C, estrous cycle; P, pregnancy; LE, luminal epithelium; GE, glandular epithelium. Bars = 100 μm and 50 μm in inset.

Next, we performed *in situ* hybridization analysis to determine which cell type(s) express *S100A7A* mRNA in the endometrium. As shown in Figure 2, the expression of *S100A7A* mRNA was primarily localized to luminal epithelial (LE) cells and superficial glandular epithelial (GE) cells in the endometrium during the estrous cycle and pregnancy with strong signal intensity in LE cells on day 12 of pregnancy. In skin tissue used as a positive control, the expression of *S100A7A* mRNA was localized to stromal and hair follicle cells.

### *S100A7A* expression in conceptuses during early pregnancy and chorioallantoic tissue during later stages of pregnancy

**Figure 3:**
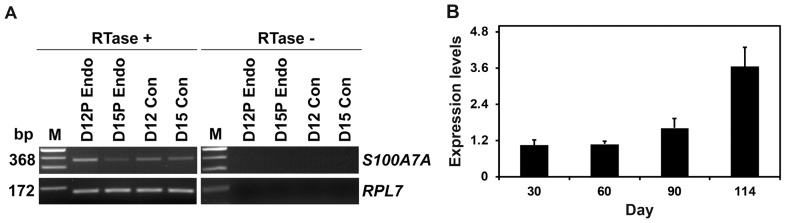
S100A calcium binding protein A7A (*S100A7A*) expression by conceptuses from day 12 and day 15 of pregnancy and by chorioallantoic tissues during late pregnancy. (A) Reverse transcription polymerase chain reaction (RT-PCR) analysis of *S100A7A* mRNA in conceptuses on days 12 and 15 of pregnancy was performed using total RNA preparations. Porcine ribosomal protein L7 (*RPL7*) was used as a positive control. (B) Real-time RT-PCR analysis of the expression of S100A7A mRNA in chorioallantoic tissue samples on days 30, 60, 90, and 114 of pregnancy. Data are reported as expression relative to that detected on day 30 of pregnancy after normalization of the transcript amount to the endogenous *RPL7*, ubiquitin B, and TATA binding protein controls, and data are presented as means with standard errors. RTase +/−, with (+) or without (−) reverse transcriptase; M, molecular marker; D12 Endo, endometrium on day 12 of pregnancy; D12 Con, day 12 conceptus; D15 Con, day 15 conceptus.

Having determined that *S100A7A* mRNA was detected in the endometrium in a stage-specific and cell type-specific manner, we then assessed whether conceptus tissues express *S100A7A* during early pregnancy. We performed RT-PCR using cDNAs from conceptuses from days 12 and 15 and detected *S100A7A* in conceptus tissues on both days of pregnancy (Figure 3A). Real-time RT-PCR analysis was performed to determine whether the expression of *S100A7A* changes in chorioallantoic tissues during pregnancy. The expression of *S100A7A* in chorioallantoic tissues during mid- to term pregnancy increased toward term pregnancy (quadratic effect of day, p<0.01) (Figure 3B).

### Effects of steroid hormones, E_2_ and P_4_, and IL1B on *S100A7A* expression in endometrial tissues

**Figure 4:**
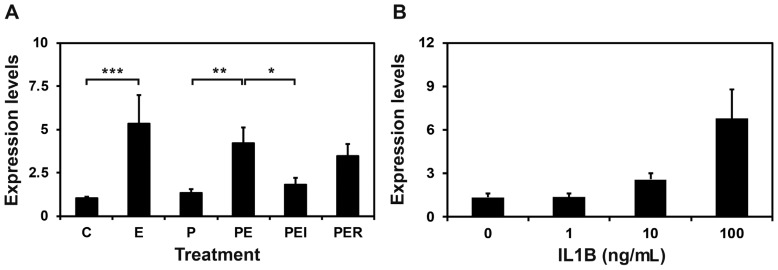
Effects of steroid hormones (A) and IL1B (B) on S100A calcium binding protein A7A (*S100A7A*) expression in endometrial explant cultures. Endometrial explants from gilts on day 12 of the estrous cycle were cultured in the presence of control (C), E_2_ (estradiol-17β; E), P_4_ (progesterone; P), E_2_+P_4_ (PE), E_2_+P_4_+ICI (I, an estrogen receptor antagonist) (PEI), or E_2_+P_4_+RU (R; a progesterone receptor antagonist) (PER) and interleukin-1β (IL1B). For each treatment, all experiments were repeated in triplicate with endometrium from each of three gilts. Abundance of mRNA expression based on real-time reverse transcription polymerase chain reaction analyses is relative to that for *S100A7A* mRNA in the control group of endometrial explants after normalization of transcript amounts to porcine ribosomal protein L7, ubiquitin B, and TATA binding protein mRNAs. Data are presented as means with standard error. The asterisks denote statistically significant difference: * p<0.05; ** p<0.01; *** p<0.001.

Next, we determined the factor(s) regulating *S100A7A* expression in the endometrium based on the results indicating that the abundance of *S100A7A* mRNA in the endometrium was greatest on day 12 of pregnancy. Because estrogen and IL1B of conceptus origin are secreted into the uterine lumen with the greatest abundance on day 12 of pregnancy and the expression of many endometrial genes is regulated estrogen and IL1B of conceptus origin and/or P_4_ from the CL in pigs [4], we hypothesized that E_2_, P_4_, and/or IL1B may affect the expression of *S100A7A* in the endometrium. As shown in Figure 4A, *S100A7A* mRNA level was increased by E_2_ (Control vs E_2_, p<0.001; P_4_ vs P_4_+E_2_, p<0.01) but not by P_4_. The E_2_-induced increase in *S100A7A* mRNA was inhibited by ICI, an estrogen receptor antagonist (P_4_+E_2_ vs P_4_+E_2_+ICI, p<0.05). IL1B also increased the expression of *S100A7A* in a dose-dependent manner in endometrial tissues (linear effect of dose, p<0.01), as shown in Figure 4B.

## DISCUSSION

The significant findings of this study indicated that: i) *S100A7A* is expressed in the endometrium during the estrous cycle and pregnancy in a pregnancy status- and stage-dependent manner, ii) *S100A7A* mRNA is localized to endometrial LE and GE with strong intensity in LE on day 12 of pregnancy, iii) early stage conceptuses and chorioallantoic tissues from day 30 to term pregnancy express *S100A7A*, and iv) E_2_ and IL1B induce *S100A7A* in the endometrium. To our knowledge, this is the first report characterizing the expression of *S100A7A* in the endometrium throughout the estrous cycle and pregnancy and regulation of *S100A7A* endometrial expression by steroid hormones and IL1B.

Although there are currently no studies on the expression and function in the endometrium in any species, we have shown that *S100A7A* is differentially expressed in the endometrium between day 12 of the estrous cycle and day 12 of pregnancy [7]. In this study, we further examined the expression of *S100A7A* throughout the estrous cycle and pregnancy. The results showed that *S100A7A* was expressed in the endometrium with the greatest abundance on day 12 during pregnancy and was maintained low during all stages of the estrous cycle in pigs. The period of increased endometrial *S100A7A* expression coincides with the time of conceptus elongation and implantation initiation in pigs. The porcine conceptuses in the uterine lumen elongate from spherical to tubular and to filamentous shapes on days 10 and 12 of pregnancy to increase the surface area for attachment to the endometrium and to initiate implantation to the endometrium on or around day 12 to form an epitheliochorial placenta [2–4]. Thus, our results suggest that endometrial *S100A7A* expression is a good marker of conceptus implantation initiation in pigs. In addition, *S100A7A* was also expressed in early-stage conceptuses and in chorioallantoic membranes during mid-to-late pregnancy, indicating that *S100A7A* may be important for conceptus development and placental formation during pregnancy.

During the implantation period, the elongating porcine conceptuses secrete various factors, including estrogen and IL1B [4]. Estrogen increases the expression of many genes such as *AKR1B1*, *FGF7*, IL1 receptor accessory protein (*IL1RAP*), *LPAR3*, *SPP1*, and *TRPV6*, and IL1B induces the expression of *ABCC4*, *ABCC9*, *AKR1B1*, *SLCO2A1*, *SLCO4C1*, and *SLCO5A1* and decreases the expression of inhibitor of DNA binding 2 in the endometrium during the implantation period [4,17]. We hypothesized that estrogen and IL1B of conceptus origin might induce the endometrial expression of *S100A7A* on day 12 of pregnancy, because the results showed that the expression of *S100A7A* in the endometrium was greatest on day 12 of pregnancy, when the conceptus secretes estrogen and IL1B into the uterine lumen [2–4]. Indeed, our data showed that both estrogen and IL1B increased the expression of endometrial *S100A7A*, suggesting that conceptus-derived estrogen and IL1B are responsible for increased expression of *S100A7A* in the endometrium during early pregnancy in pigs. In human skin, IFN-γ increases *S100A7A* expression [11], and it is well known that the implanting porcine conceptus secretes a large amount of IFN-γ into the uterine lumen during early pregnancy, with the greatest amount on days 15 and 16 [18]. Because endometrial expression of *S100A7A* was reduced on day 15 of pregnancy in this study, it is likely that IFN-γ does not affect the expression of *S100A7A* in the endometrium in pigs.

This study showed that the expression of *S100A7A* was epithelial-specific in the endometrium, especially in LE and superficial GE cells. It is well established that endometrial epithelial cells lose PGR during the diestrus stage of the estrous cycle and in the corresponding period during pregnancy in most mammalian species [19]. In pigs, PGR in LE and GE cells declines from day 7 and is undetectable in LE and superficial GE cells on day 12 of the estrous cycle and pregnancy [20,21]. In comparison, endometrial estrogen receptor-α (ESR1) increases from day 0 to day 12 of the estrous cycle and pregnancy, with the highest level between days 10 and 12 [22]. Since the presence of PGR and ESR1 is the same in the endometrium between day 12 of the estrous cycle and day 12 of pregnancy, it seems that the difference of endometrial epithelial *S100A7A* expression between day 12 of the estrous cycle and pregnancy is due to conceptus-derived estrogen. Furthermore, because estrogen increases the expression of *IL1RAP*, a subunit of the IL1B receptor, and IL1B induces the expression of IL1 receptor 1 (*IL1R1*), the other subunit for the IL1B receptor, in the endometrium in pigs [5], it seems that there is a cooperative action of conceptus-derived estrogen and IL1B for endometrial epithelial *S100A7A* expression through induction of *IL1R1* and *IL1RAP* expression during the implantation period.

During the implantation period in pigs, endometrial cal cium release into the uterine lumen increases significantly as the conceptus elongates, and estrogen of conceptus origin is responsible for calcium secretion [4]. At the same time, the endometrium in response to estrogen induces the expression of many calcium-related molecules such as *S100G*, *SPP1*, stanniocalcin 1, and *TRPV6* [4]. *TRPV6* and *S100G* regulate epithelial calcium absorption in the endometrium [23,24], and *SPP1* plays an important role in cell-to-cell adhesion between the conceptus trophectoderm and endometrial epithelial cells [2]. Because the implantation process requires many cell adhesion molecules such as integrins, cadherins, selectins, and *SPP1*, and the calcium ion is important for this process, it has been hypothesized that the calcium ion is regulated by conceptus-derived estrogen at the maternal-conceptus interface and is mainly involved in cell adhesion between the trophectoderm and epithelial cells. Results of this study showed that *S100A7A*, a member of the S100 protein family of calcium binding proteins, was expressed in the endometrial epithelial cells in response to conceptus-derived estrogen and IL1B. *S100A7A* is involved in various biological processes, including innate immunity, antimicrobial function, and epithelial tumorigenesis [10,12,25]. Thus, our results suggest that calcium ions at the maternal-conceptus interface during the implantation period may regulate not only cell-to-cell adhesion, but also other cellular functions, although the detailed function of *S100A7A* in the endometrium at the time of implantation in pigs is not fully understood.

In conclusion, the results of this study in pigs showed that *S100A7A* was expressed in the endometrial epithelial cells with the greatest abundance on day 12 of pregnancy, which corresponds to the time of initiation of conceptus implantation, induced by estrogen and IL1B of conceptus origin. This could be a unique endometrial epithelial marker of conceptus implantation in pigs. These findings provide an important insight into the understanding of conceptus-endometrial interactions for the successful establishment of pregnancy in pigs.

## Figures and Tables

**Table 1:** Summary of primer sequences for real-time RT-PCR and RT-PCR and expected product sizes

Primer	Sequence of forward (F) and reverse (R) primers (5′ → 3′)	Annealing temperature (°C)	Product size (bp)	GenBank accession no.
For RT-PCR				
*S100A7A*	F: TTG CCG AGA CTC CAT AGT CC	60	221	ENSSSCG00000006591
	R: CTT GCC ACA GAC ACA CAA GG			
*RPL7*	F: AAG CCA AGC ACT ATC ACA AGG AAT ACA	60	172	NM_001113217
	R: TGC AAC ACC TTT CTG ACC TTT GG			
For real-time PCR				
*S100A7A*	F: GCA GAC AAG GAC AAG GAC AAC	60	368	NM_001113217
	R: GGA AGG ACA GAC GTG AAA GC			
*RPL7*	F: AAG CCA AGC ACT ATC ACA AGG AAT ACA	60	172	NM_001113217
	R: TGC AAC ACC TTT CTG ACC TTT GG			
For *in situ* hybridization				
*S100A7A*	F: AGC ACT GCC CCT GTC CTG	60	374	ENSSSCG00000006591
	R: CCG TGC TCC CTC TAA TAA AGA C			

RT-PCR, reverse transcription polymerase chain reaction; *S100A7A*, S100A calcium binding protein A7A; *RPL7*, ribosomal protein L7.
